# Organic Farming Improves Pollination Success in Strawberries

**DOI:** 10.1371/journal.pone.0031599

**Published:** 2012-02-15

**Authors:** Georg K. S. Andersson, Maj Rundlöf, Henrik G. Smith

**Affiliations:** 1 Centre for Environmental and Climate Research, Lund University, Lund, Sweden; 2 Department of Biology, Lund University, Lund, Sweden; 3 Department of Ecology, Swedish University of Agricultural Sciences, Uppsala, Sweden; University College London, United Kingdom

## Abstract

Pollination of insect pollinated crops has been found to be correlated to pollinator abundance and diversity. Since organic farming has the potential to mitigate negative effects of agricultural intensification on biodiversity, it may also benefit crop pollination, but direct evidence of this is scant. We evaluated the effect of organic farming on pollination of strawberry plants focusing on (1) if pollination success was higher on organic farms compared to conventional farms, and (2) if there was a time lag from conversion to organic farming until an effect was manifested. We found that pollination success and the proportion of fully pollinated berries were higher on organic compared to conventional farms and this difference was already evident 2–4 years after conversion to organic farming. Our results suggest that conversion to organic farming may rapidly increase pollination success and hence benefit the ecosystem service of crop pollination regarding both yield quantity and quality.

## Introduction

Agricultural intensification, resulting in loss and degradation of natural and semi-natural habitats, threatens biodiversity [1], [2], [3] and associated ecosystem services [4], [5]. Pollination provided by wild pollinators can increase crop production and benefit wild plants [6], though recent declines in pollinator numbers has been suggested as one reason for pollination deficiency in crops ([6], [7] but see [8]). Although the major crops are not dependent on pollinators, large proportions of human nutrient supply come from pollinator dependent crops [9]. Hence it is important to understand how pollinators and pollination in the agricultural landscapes can be enhanced.

Organic farming has been proposed to be a means to alleviate the decreasing biodiversity in agricultural landscapes [10], [11], [12]. It mainly differs from conventional farming by the prohibition of most pesticides and inorganic fertilisers [13], necessitating more elaborate crop-rotations such as the use of nitrogen-fixing plants [14]. Organic farming has been shown to affect biodiversity of several taxonomic groups [15], [16], [17], [18], but effect strength and direction can vary with taxonomic group [19], scale [12] and landscape context [16]. However, current results suggest that management effects benefiting biodiversity may not necessarily translate into improved ecosystem services [20].

Pollinator diversity and abundance often benefit from organic farming [21], [22], [23], which may lead to improved pollination [17], [24], [25]. However, how pollination is influenced by farming practice is not thoroughly understood and studies have generated contrasting results [7], [26], [27], [28]. Effects of organic farming on pollination are not necessary simply related to pollinator richness or abundance as pollination rates may be modified by e.g. community composition, visitation frequencies, and foraging behaviour. Therefore it is important to first understand if the effects of organic farming on pollinator diversity and abundance translate into enhanced crop pollination, and then further examine the causes.

Past land-use can affect biodiversity during shorter or longer transition periods [29], [30], suggesting that there may be a time-lag between land-use change and resulting changes in biodiversity and abundance [30], [31]. The time-lag effect can have two main causes. First, time-lags may arise as a consequence of that organisms respond slowly to changes in habitat extension, distribution and quality, e.g. by slow dispersal, slow reproduction or both [32]. Second, diversity can respond to physical properties of the environment that change slowly themselves under the new regime, e.g. slow build-up of carbon in soil or formation of new patches of habitats. For example, a recent study showed such temporal patterns for transition time to organic farming on butterfly abundance [33]. This was attributed to a possible low carrying capacity of arable land for butterflies and a hence a steady slow increase in abundance over years with habitat improvement. Hence it can be hypothesized that effects of organic farming on pollination may not immediately be evident after conversion to organic farming. To our knowledge, this hypothesis has not been empirically tested.

We studied the effects of transition from conventional to organic farming on the pollination of strawberries, a crop that benefits from biotic pollination[34], [35], [36] and is visited by a wide range of pollinators, e.g. hoverflies and wild bees [37]. The main questions were (*i)* is the pollination success in strawberries higher on organic compared to conventional farms and (*ii)* is there a time-lag effect in pollination success since transition to organic farming.

## Methods

All necessary permits were obtained for the described field studies. The farmers, on whose properties we conducted the study, were informed about, and approved, the studies before they started. The study was conducted on 12 farms in Scania, the southernmost part of Sweden, in 2009. Circular landscapes with a radius of 1 km around farms were digitized using ArcGIS 9.2 and characterized with information from the Integrated Administration and Control System, IACS, a database for agricultural land-use in Sweden administrated by the Swedish Board of Agriculture. We described land-use intensity using an index calculated as the proportion of annual crops and ley of all farmland and forest within the circular landscape, excluding e.g. urban areas and lakes. The index therefore reflects the proportion of the landscape under intensive cultivation. To avoid bias, in terms of organic farming being more common in complex landscapes [16], we selected all farms in landscapes where the proportion of intensively cultivated land was at least 70%.

The twelve farms were either conventionally managed (n = 4), recently transformed to organic farming (“new”, 2–4 years; n = 4) or under organic management for a longer time period (“old”, 14–24 years; n = 4). The organic farms were certified according to the Swedish certification organisation for organic products, KRAV, which mainly follows the European Council Regulation [13]. To assess pollination we established strawberry (*Fragaria ananassa*) plants as phytometers which we placed in 7.5 litre pots adjacent to field borders of spring wheat or barley to minimize the crop influence on pollinators. We placed the pots halfway down in the ground to reduce water loss and watered them when necessary. This method makes it possible to control for potential confounding factors such as soil-type. To make cross-pollination possible four pots of strawberries were used at each farm and placed just far enough to prevent the flowers to come into physical contact with each other. We used strawberries for two main reasons. First, a strawberry is an aggregated fruit where the pollination success can be assessed on each strawberry. This means that pollination success can be measured independent of total fruit set and thereby all variables apart from pollination success that may influence total fruit set. Strawberry pollination success is also increased by insect pollination [34], [35], [36]. Second, strawberries are an economically important crop in Sweden. We further address these issues in the discussion.

The phytometers were placed outside between June 2^nd^ and August 8^th^, to allow visitation of native pollinators in the study landscapes. None of our farms had managed honeybees on them. The flowering period lasted from June 5^th^ to July 3^rd^ and all fully ripe strawberries were continuously collected. Pollination success was estimated by counting the numbers of malformations on each strawberry and the proportion of fully pollinated berries per farm. These malformations are formed when ovaries are not fertilised. The area of the receptacle holding these ovaries does then not swell up which results in a malformed strawberry [36]. The malformations we counted are thus areas of unfertilized achenes, or seeds, on the mature strawberry corresponding to the unpollinated stigmas and unfertilised ovaries, on the flower. On average 18.9±2.1 (SE) strawberries per farm were collected and counted for malformations.

We analysed pollination success (mean number of miss-formation and proportion fully pollinated strawberries) in relation to farming practice (conventional vs. organic) and in relation to age category (new organic vs. old organic) in R ver. 2.13.1 [38]. First, the overall difference between the three categories was tested in an ANOVA. Then we used *a priori* contrasts to test, first the difference between conventional and organic farming, and second the difference between new and old organic farms. To test the proportion of fully pollinated strawberries we used a generalized linear model assuming binomial error followed by same *a priori* contrast tests as above.

## Results

**Figure 1:**
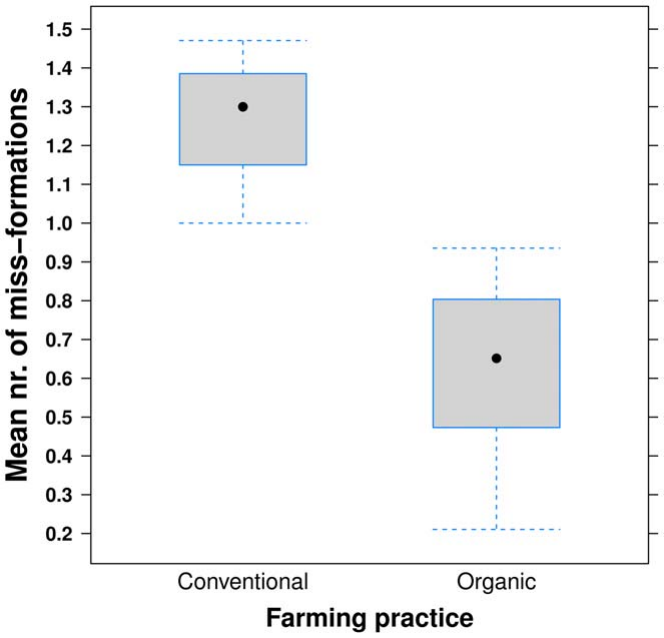
Mean number of malformations. The mean number of malformations on strawberries from plants on organic and conventional farms. Boxes represent 25^th^ and 75^th^ of the sample, dots the median and error-bars the minimum and maximum values respectively.

**Figure 2:**
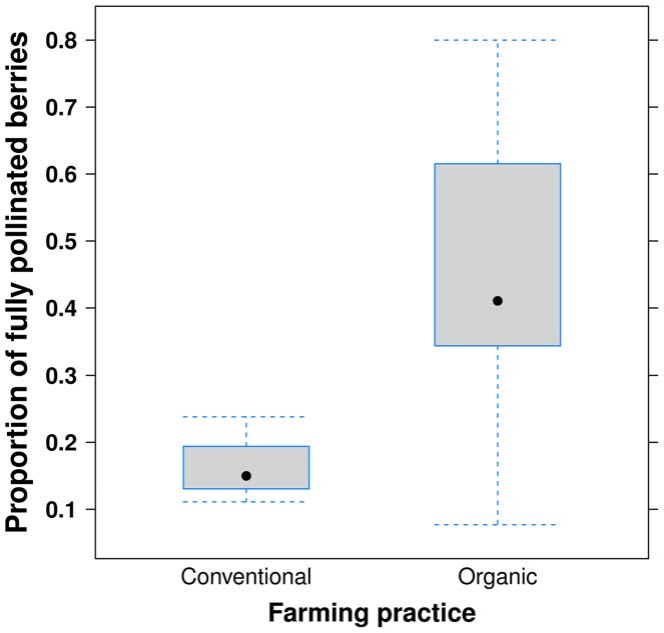
Proportion of fully pollinated strawberries. The proportion of fully pollinated strawberries, i.e. having no malformations, from plants on organic and conventional farms. Boxes represent 25^th^ and 75^th^ of the sample, dots the median and error-bars the minimum and maximum values respectively.

The global ANOVA model showed a significant effect of farming practice (conventional, new organic, old organic) on mean number of malformations (F_2,8_ = 7.5, p = 0.015). The mean number of malformations on strawberries was lower on organic farms (0.63±0.089; mean ± SE), compared to conventional farms 1.26±0.14 (t_8_ = −3.74, p = 0.0057; Fig. 1). There was also an effect of farming practice on the proportion of fully pollinated berries (z_10_ = 2.86, p = 0.0043) with a higher proportion of fully pollinated berries on organic farms (0.45±0.081) compared to conventional farms 0.17±0.037 (z_10_ = 2.77, p = 0.0055; Fig. 2). The number of malformations did not differ between new (0.71±0.08) and old (0.54±0.16) organic farms (t_8_ = −0.96, p = 0.37), nor did the proportion fully pollinated berries, 0.40 and 0.51 on new and old organic farms, respectively (z = −1.14, p = 0.26).

## Discussion

Pollination potential in strawberries was significantly higher on organic compared to conventional farms, shown by the fewer malformations on berries and a higher proportion of fully pollinated berries. However, we found no effect of time since transition to organic farming on either the number of malformations or the proportion of fully pollinated strawberries. This suggests that the increase in pollination success occurs already within a few years after conversion to organic farming. As butterfly and plant species richness has been found to increase rapidly after transition to organic farming [33], our result suggest that pollinator richness may respond rapidly as well.

The few studies previously examining the effect of organic farming on seed-set, some of which has been on wild plants [17], [39], have found varied results. Turnip rape-seed on organic farms had less pollination deficit than rape-seed on conventional farms [27] whereas seed-set in petunias did not differ between farming practices [26]. Strawberries, as an aggregate fruit, allowed us to assess pollination on each individual berry and consequently compare the actual pollination success between farms. A caveat when estimating pollination success on plants with non-aggregate fruits is that a number of factors other than pollination can influence seed set. Non-aggregated fruits, or simple fruits, can have many seeds but they come from a flower with only one pistil. Thus, as each seed can be affected by predation, be aborted or just not develop for various reasons, it is harder to relate the number off seeds in such types of fruits to the pollination success. Aggregate fruits come from one flower with many pistils where every ovary forms one seed, in strawberries an achene. This has the advantage that, as in the case of the strawberries, each individual strawberry, shows the pollination success. In our study 45% of the strawberries were fully pollinated on organic farms with only 17% being fully pollinated on conventional ones. In one earlier study [7] watermelons were found to receive sufficient pollen deposition to allow production of fully developed fruits on every second organic farm, but not on any conventional farm. However, no significant difference between farming practices in pollination services remained after accounting for isolation from natural habitats [7]. In the present study any confounding landscape factors were explicitly controlled for in the design of the field experiments.

Possible explanations for the higher pollination success may be a higher abundance, visitation frequency or a higher diversity of pollinators, which has been shown to be supported by organic farms [22], [23]. Several studies suggest a correlation between pollinator diversity and fruit-set [24], [40], [41]. However, in strawberries, the influence of farming practice on pollination success may not result from a higher diversity of pollinators *per se* at organic farms, but may be a consequence of community composition of pollinators [7]. Chagnon et al. [42] showed that the quality of strawberries is affected by the composition of pollinator communities, with large- and average-sized apoids pollinating the top and small-sized apoids pollinating the bottom and sides of strawberries. However, single visits by honeybees, large hoverflies and small solitary bees contributed equally to pollination of strawberries [34] indicating that no group alone is most important. Based on our results it remains to be tested if there is a higher number of functionally important pollinator groups at organic farms or if other explanations, such as an increase in pollinator abundance, can account for the increased pollination success.

Our results suggest that increased pollination success mediated by organic farming may increase both crop yield quantity and quality on farms growing strawberries. This is economically important since approximately 11 000 tonnes of strawberries are produced annually in Sweden according to Swedish board of Agriculture statistics, 2011 and 40 million tonnes globally [43]. However, since we use phytometers, our results are not directly related to estimates of differences in crop pollination at a farm scale. First, we did not determine if strawberry plants compensate for low pollination success by e.g. producing more berries. Second, the effects may be scale-dependent hence farms with whole fields of strawberries the pattern in relation to farming practice may be different compared to a limited number of strawberry plants. In order to link our results to production in strawberry fields, simultaneous measurements of pollination in both fields and phytometers will be required.

To further determine the relationship between farming practice and the functions pollinators provide enhances our understanding of measures needed to preserve pollinators. Although the mechanistic cause for the observed differences in pollination success between farming practices remains unknown in our study, effects were present at recently transformed farms. Combined with results on other ecosystem services, our results imply that agri-environment schemes should be evaluated both directly after implementation and over a longer term.
